# PHF14: an innate inhibitor against the progression of renal fibrosis following folic acid-induced kidney injury

**DOI:** 10.1038/srep39888

**Published:** 2017-01-03

**Authors:** Bo Yang, Sixiu Chen, Ming Wu, Lin Zhang, Mengna Ruan, Xujiao Chen, Zhengjun Chen, Changlin Mei, Zhiguo Mao

**Affiliations:** 1 Kidney Institute of CPLA, Division of Nephrology, Changzheng Hospital, Second Military Medical University, Shanghai, 200003, People’s Republic of China; 2 School of Life Science and Technology, Shanghai Tech University, Shanghai 200031, People’s Republic of China; 3 State Key Laboratory of Molecular Biology, Institute of Biochemistry and Cell Biology, Shanghai Institutes for Biological Sciences, Chinese Academy of Sciences, Shanghai 200031, People’s Republic of China

## Abstract

PHF14 is a newly identified regulator of mesenchyme growth in embryonic tissues. Previous studies have shown that phf14-null mutants die just after birth due to interstitial tissue hyperplasia in major organs, including the kidneys. The aim of this study was to investigate PHF14 function in renal fibrosis. By studying the chronic kidney injury mouse model, we found that PHF14 was upregulated in fibrotic kidneys after renal insults induced by folic acid administration. Compared with wild-type mice, PHF14-null mice showed more severe renal fibrosis after pro-fibrotic stimuli. Moreover, PHF14 in rat renal fibroblasts was upregulated by transforming growth factor-β (TGF-β) stimulation; while this upregulation was inhibited when smad3 phosphorylation was blocked. A chromatin immunoprecipitation (ChIP) assay further indicated that phospho-smad3 (p-smad3) acted as a transcription factor to enhance PHF14 expression. A lack of PHF14 expression enhanced collagen I and α-smooth muscle actin (α-SMA) synthesis induced by TGF-β *in vitro*. PHF14 was involved in inhibition of platelet-derived growth factor (PDGF) signaling overactivation by selectively repressing PDGF receptor-α (PDGFR-α) transcription. In summary, PHF14 expression was upregulated in fibrotic models *in vivo* and *in vitro*, and the TGF-β/smad3/PHF14 pathway acted as a self-limiting mechanism in the TGF-β-dominated renal pro-fibrotic process by suppressing PDGFR-α expression.

Progressive kidney tubulointerstitial fibrosis (TIF) is the major pathological basis and the common pathway of all chronic kidney diseases, eventually leading to uremia^1^,^2^. After severe initial insults, failed wound healing in the kidney is the main event underlying progressive TIF^3^,^4^. In clinical settings, some patients who survive episodes of acute kidney injury will still progress to chronic kidney disease in the following decades after injury^5^,^6^, implying that the progression of TIF is triggered by severe kidney insults.

The process of renal fibrogenesis is mediated by multiple mediators^7^, among which transforming growth factor-β (TGF-β) is widely recognized as a crucial factor^8^,^9^,^10^. The imbalance between the pro-fibrotic effects and the counter-regulatory effects of TGF-β hinders kidney tissue repair, interrupts tissue homeostasis, and initiates the process toward renal fibrosis^11^,^12^,^13^. However, the complicated biological network of these mediators has not been fully elucidated.

PHF14, a newly identified mesenchyme growth regulator^14^, is highly conserved from *Caenorhabditis elegans* to humans. PHF14 can bind to histones via its plant homology domain finger and is considered as a transcriptional regulator of target gene expression^15^,^16^. Previous studies have shown that PHF14 works as a platelet-derived growth factor receptor-α (PDGFR-α) transcription factor by suppressing the transcription of PDGFR-α heterogeneous nuclear RNA and that phf14-null mice die just after birth due to significant interstitial hyperplasia and fibrosis in major organs (e.g., lung, liver, and kidney)^14^,^17^. Based on previous research, we hypothesized that PHF14 might play a role in renal tissue repair and regeneration during the progression of fibrosis after pro-fibrotic insults. Understanding the function of PHF14 will help to elucidate the interactions of mediators in the process of renal fibrogenesis. Therefore, the primary objectives of this study were to investigate the expression profile of PHF14 in the scenario of TIF following acute kidney injury induced by folic acid, to illuminate the role of PHF14 in renal TIF, and to examine the interaction between PHF14 and TGF-β signaling.

## Results

### PHF14 is upregulated in a folic acid-induced kidney fibrosis mouse model

**Figure 1:**
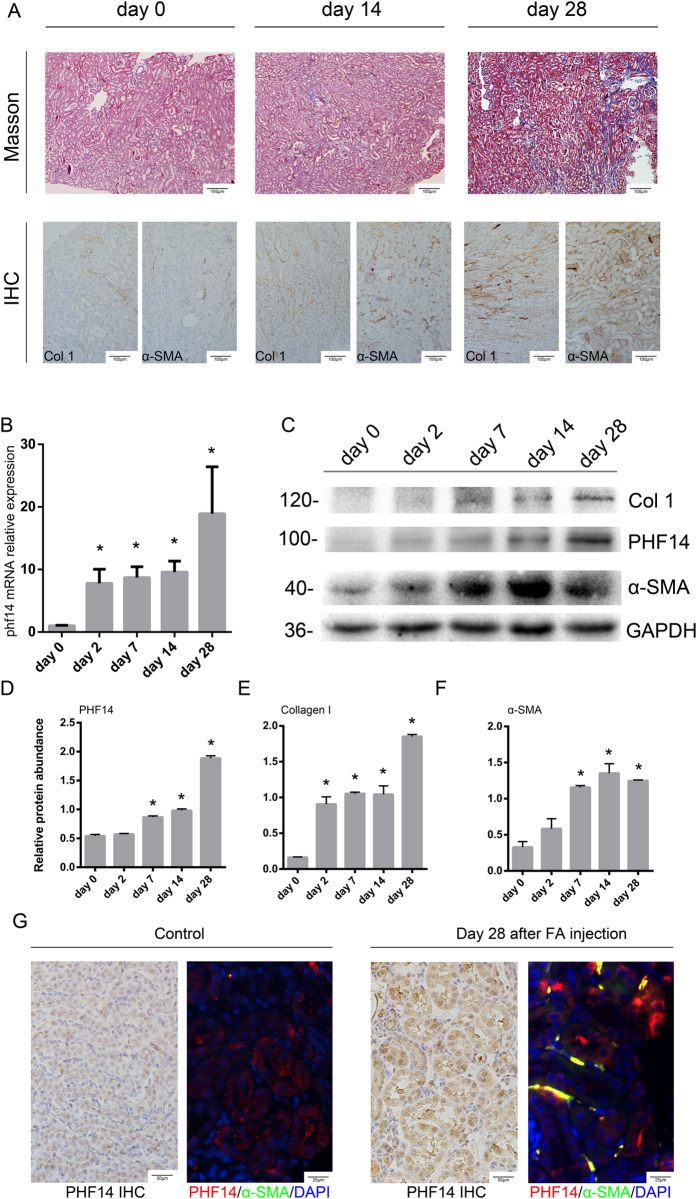
Activation of PHF14 expression in the kidneys after pro-fibrotic insult. (**A**) Representative Masson and immunohistochemical staining assay images demonstrating the progression of fibrosis after folic acid administration. (**B**) Quantitative PCR (Q-PCR) analysis showing persistent elevation of PHF14 expression in the kidney on day 2, day 7, day 14, and day 28 after folic acid administration, compared with the sham control. (**C**) Western blot results showing that PHF14 protein expression increased with the progression of fibrosis. Anti-GAPDH was used to verify equivalent loading. (**D**–**F**) Semiquantitative analysis of PHF14, collagen I, and α-SMA protein abundance in the kidneys. (**G**) Representative immunohistochemical/immunofluorescent staining images demonstrating the elevation of PHF14 protein in fibrotic kidneys on day 28 after folic acid administration, compared with the sham control. Immunofluorescence images revealed the co-localization of elevated PHF14 and α-SMA expression in activated myofibroblasts. *P < 0.05, compared with sham controls (n = 5), Col 1, collagen I; α-SMA, α-smooth muscle actin; GAPDH, glyceraldehyde 3-phosphate dehydrogenase; IHC, immunohistochemistry; DAPI, 4′,6-diamidino-2-phenylindole; FA, folic acid.

To explore the expression profile of PHF14 after pro-fibrotic insults in mice, a folic acid-induced kidney fibrosis mouse model, a well characterized model of TIF following acute kidney injury, was used for this study. Mice were euthanized at predesigned acute and subsequent chronic phases after folic acid administration^18^,^19^. Masson and immunohistochemical stainings of collagen I and α-smooth muscle actin (α-SMA) in kidney sections on day 14 and day 28 after folic acid injection confirmed the successful production of the folic acid-induced kidney fibrosis model (Fig. 1A). Compared with sham controls, PHF14 mRNA and protein expression was upregulated persistently in the kidneys both in the acute and chronic phases of fibrosis in mice after folic acid injection (Fig. 1B–F). Immunohistochemical stainings of kidney sections on day 28 after folic acid or vehicle administration showed strong expression of PHF14 in renal tubular epithelial cells and interstitial cells but very weak staining of PHF14 in the sham control group. Immunofluorescence staining also demonstrated that PHF14 was increased in the fibrotic kidney and suggested its co-localization with α-SMA in myofibroblasts (Fig. 1G).

### TGF-β stimulation induces the upregulation of PHF14 *in vitro*

**Figure 2:**
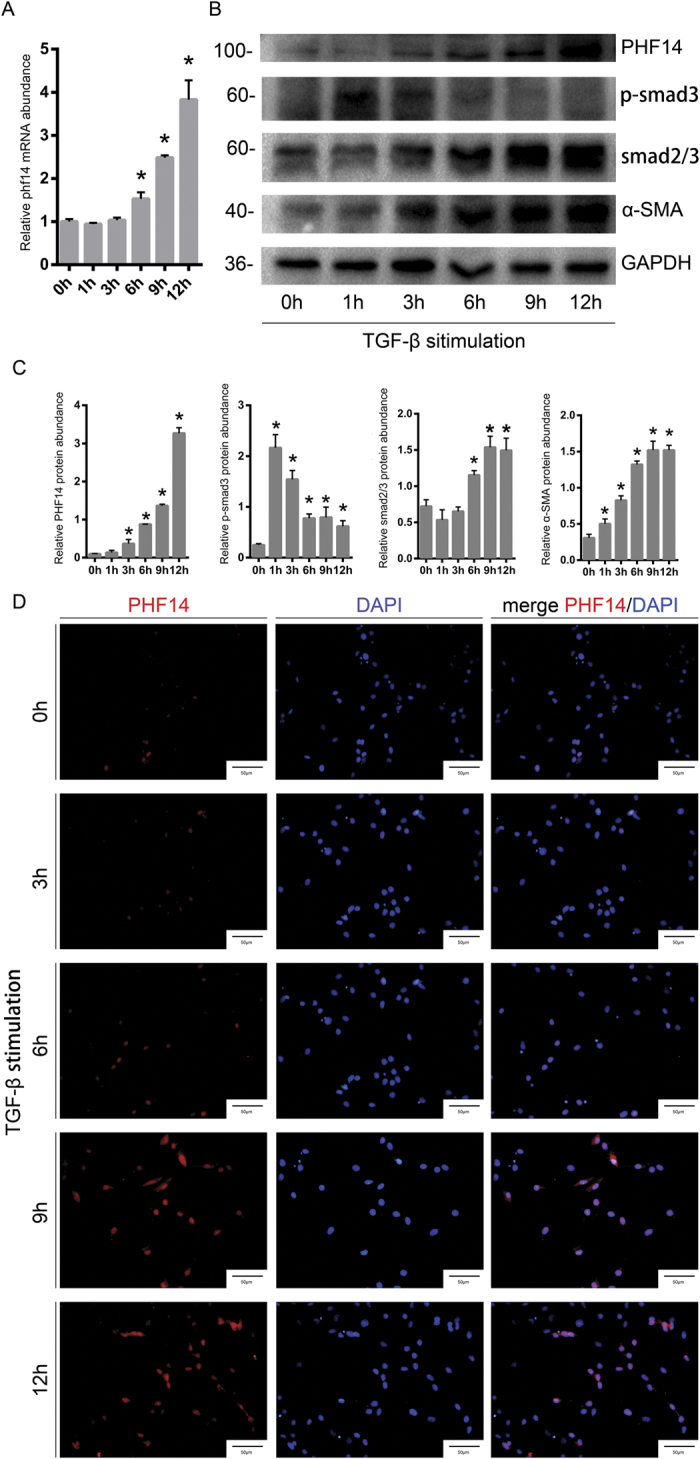
TGF-β upregulates PHF14 expression in NRK-49F cells. NRK-49F cells were treated with TGF-β (1 ng/mL) for the indicated time periods. Quantitative PCR (Q-PCR) analysis (**A**) and western blot results (**B**) demonstrating the increased expression of PHF14 protein for various periods of time. Anti-GAPDH was used to verify equivalent loading. (**C**) Semiquantitative analysis of protein abundances in the treated cells. (**D**) Immunofluorescence staining revealing the PHF14 protein expression profile in TGF-β-treated NRK-49F cells. Nuclei were visualized with DAPI (high-power field). *P < 0.05, compared with NRK-49F cells treated with TGF-β for 0 h. α-SMA, α-smooth muscle actin; TGF-β, transforming growth factor-β; p-smad3, phospho-smad3; GAPDH, glyceraldehyde 3-phosphate dehydrogenase; DAPI, 4′,6-diamidino-2-phenylindole.

To explore the association between PHF14 expression and TGF-β signaling, rat renal fibroblast (NRK-49F) cells were incubated in serum-free medium or in the presence of TGF-β (1 ng/mL). The time course of the PHF14 level and the main factors in the TGF-β signaling pathway were assessed in TGF-β-treated NRK-49F cells. After TGF-β treatment, we detected the expected increased levels of p-smad3, smad2/3, and α-SMA as well as the remarkably elevated mRNA and protein levels of PHF14 (Fig. 2A–C). By immunofluorescence staining, PHF14 was shown to be localized mainly in the nuclei, and PHF14 expression was increased after TGF-β treatment (Fig. 2D).

### P-smad3 acts as a transcription factor to enhance PHF14 expression

**Figure 3:**
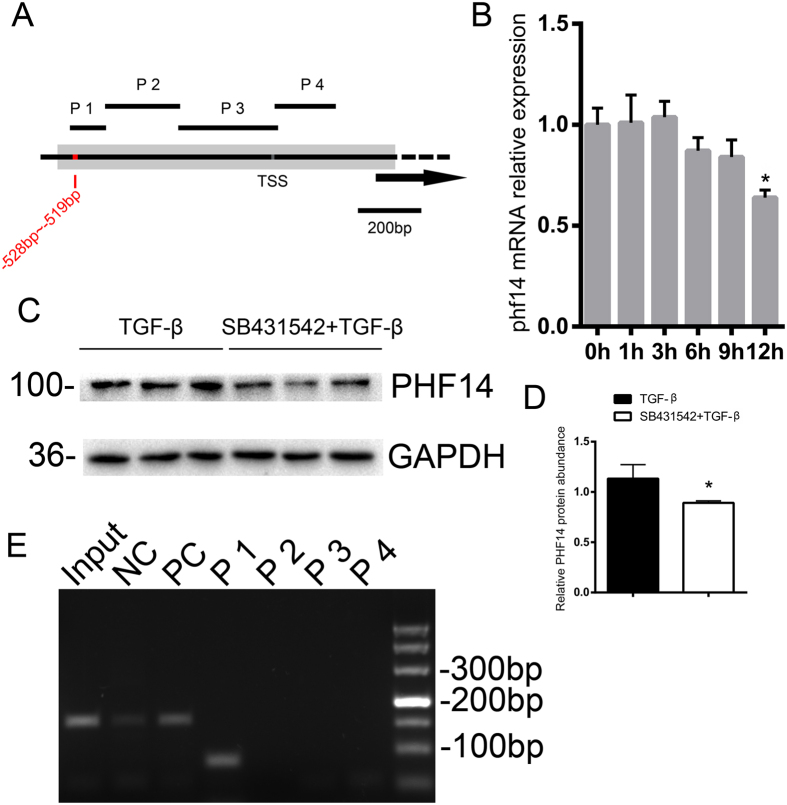
TGF-β upregulates PHF14 expression through the TGF-β/phosphorylated smad2/3 pathway. NRK-49F cells were pretreated with the TGF-β receptor 1 inhibitor SB431542 (0.6 μM) or serum-free medium for 30 min before TGF-β stimulation. (**A**) The schematic diagram of the PHF14 promoter region and the primer design. The region from -528 bp to -519 bp is the potential binding site of the smad3/4 complex predicted by virtual laboratory PROMO. (**B**) Quantitative PCR (Q-PCR) analysis showing that the otherwise elevated PHF14 mRNA level under TGF-β stimulation in the presence of SB431542 was suppressed. *P < 0.05, compared with the PHF14 mRNA level at baseline (0 h) (n = 3). (**C**) Western blot analysis demonstrating the PHF14 expression level under TGF-β stimulation in cells pretreated with SB431542, compared with NRK-49F cells pretreated with serum-free medium. Anti-GAPDH was used to verify equivalent loading. (**D**) Semiquantitative analysis of PHF14 protein abundance. *P < 0.05, compared with scrambled siRNA controls (n = 3). (**E**) Chromatin immunoprecipitation of the PHF14 promoter region with anti-p-smad3 antibody. P1–P4, primers 1 to 4; TSS, transcription start site; GAPDH, glyceraldehyde 3-phosphate dehydrogenase; TGF-β, transforming growth factor-β; NC, negative control; PC, positive control.

On the upstream promoter region of phf14, potential transcriptional factors related to TGF-β signaling were screened using virtual laboratory PROMO^20^ with the TRANSFAC database, version 8.3^21^. There was a potential binding site of smad3/4 at -528 bp to -519 bp upstream of the phf14 transcription start site (Fig. 3A).

First, we examined whether inhibiting phosphorylation of smad3 would suppress PHF14 expression. We pretreated NRK-49F cells with the selective TGF-β receptor I kinase inhibitor SB431542 (0.6 μM) or serum-free medium for 30 min before TGF-β stimulation and then extracted mRNA and protein at 12 h after TGF-β stimulation. The data showed that PHF14 upregulation was suppressed by SB431542 (Fig. 3B–D).

We further determined whether the promoter region of phf14 is directly associated with p-smad3 by using a chromatin immunoprecipitation (ChIP) assay. We found that endogenous p-smad3 was bound to the proximal upstream region of the phf14 transcription start site (Fig. 3E), indicating that phf14 might be a transcriptional target of TGF-β/smad3 signaling.

### Lack of PHF14 expression enhances collagen I and α-SMA synthesis induced by TGF-β *in vitro*

**Figure 4:**
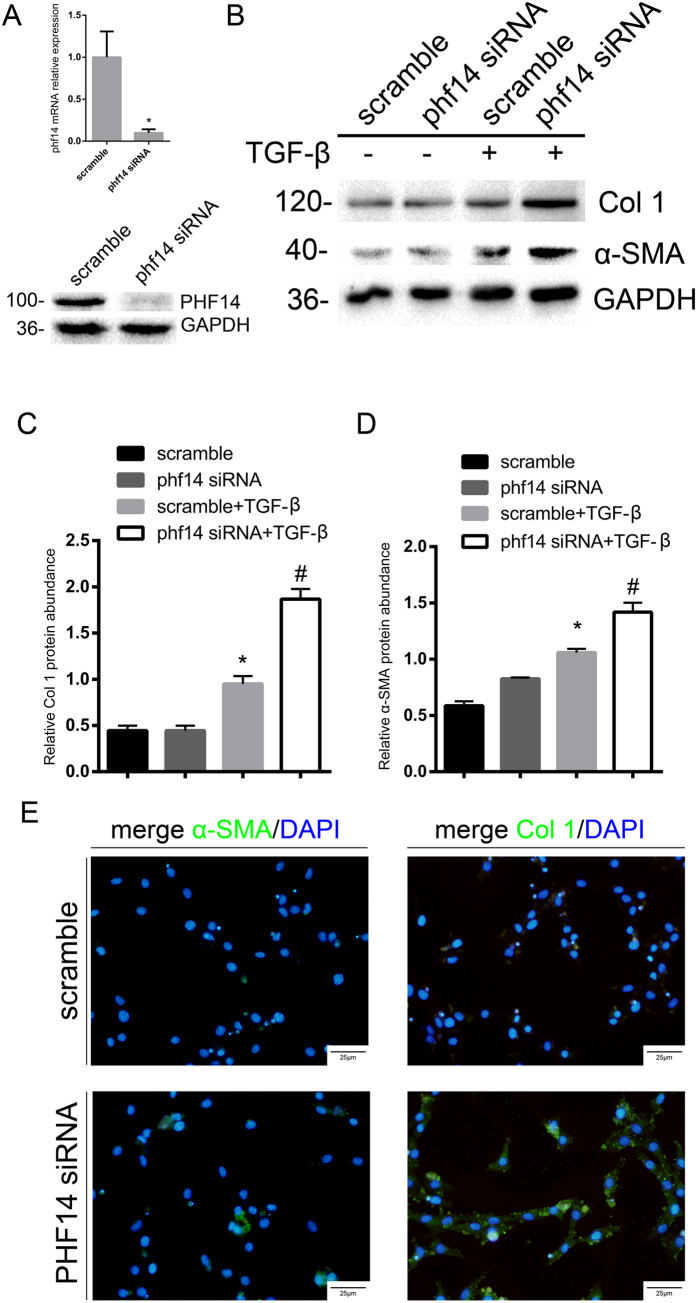
PHF14 knockdown enhances collagen I and α-SMA synthesis induced by TGF-β in NRK-49F cells. NRK-49F cells were pretreated with scrambled or PHF14 siRNA for 36 h. (**A**) Quantitative PCR (Q-PCR) demonstrating the decreased transcriptional activity of PHF14 mRNA (upper panel) and western blot analysis showing the siRNA-mediated knockdown of PHF14 (lower panel). (**B**) Western blot results demonstrating the changes of collagen I and α-SMA synthesis in the PHF14-knockdown group under conditions of TGF-β stimulation. Anti-GAPDH was used to verify equivalent loading. (**C**,**D**) Semiquantitative analysis of collagen I and α-SMA protein abundance in the TGF-β-treated NRK-49F cells. (**E**) Immunofluorescence staining revealing collagen I and α-SMA protein expression regulation in TGF-β-treated NRK-49F cells. Nuclei were visualized with DAPI (high-power field; 400 × magnification). *P < 0.05, compared with NRK-49F cells treated with vehicle; ^#^*P* < 0.05, compared with NRK-49F cells pretreated with scrambled control siRNA and cultured in the media with TGF-β (n = 3). GAPDH, glyceraldehyde 3-phosphate dehydrogenase; TGF-β, transforming growth factor-β; α-SMA, α-smooth muscle actin; Col 1, collagen I; DAPI, 4′,6-diamidino-2-phenylindole.

To delineate the role of PHF14 in TGF-β-induced fibrosis, transient silencing of PHF14 *in vitro* with PHF14 siRNA was performed in NRK-49F cells, with scrambled siRNA as a control. Knockdown of PHF14 was confirmed by quantitative polymerase chain reaction (PCR) and western blot experiments (Fig. 4A). Compared with the control group, cells lacking PHF14 expression showed significantly increased expression of collagen I and α-SMA under TGF-β stimulation (Fig. 4B–D). Immunofluorescence also showed that α-SMA and collagen I expression induced by TGF-β was increased with PHF14 knockdown, compared with the scrambled siRNA control (Fig. 4E). On the other hand, α-SMA and collagen I expression induced by TGF-β was not affected by overexpression of PHF14 with phf14-3-FLAG adenovirus infection (data not shown), suggesting that the endogenous expression levels of PHF14 were enough to maintain the normal physiological function^14^.

### PHF14-containing complex inhibits platelet-derived growth factor (PDGF) signaling overactivation by selectively repressing PDGFR-α transcription

**Figure 5:**
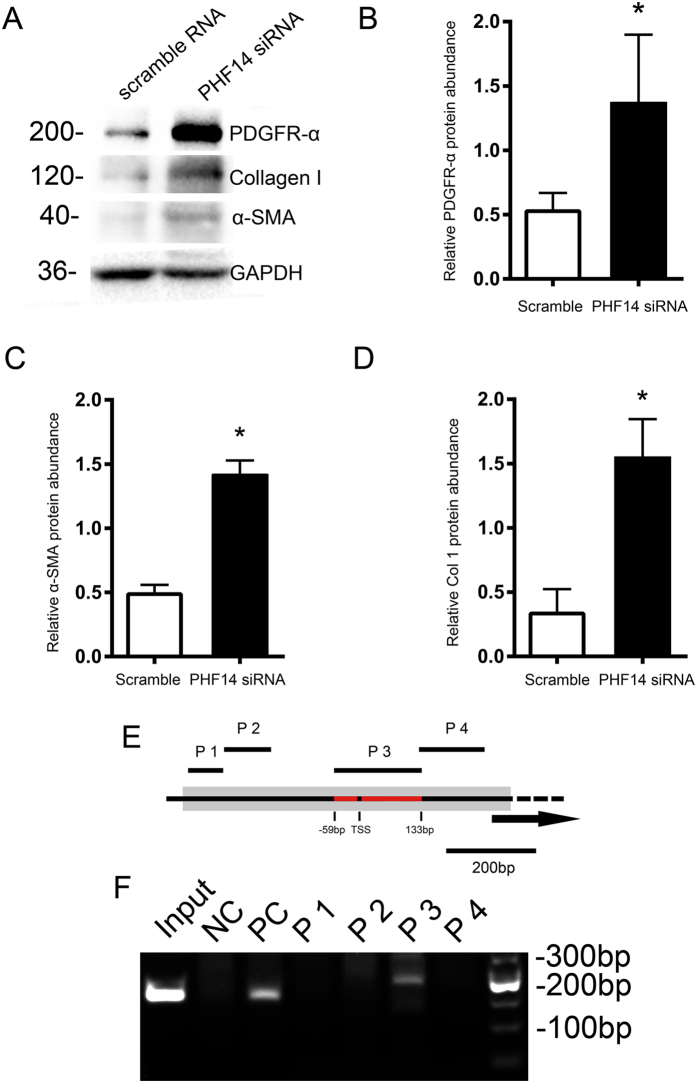
PHF14 directly represses the transcription of PDGFR-α. NRK-49F cells pretreated with scrambled or PHF14 siRNA were stimulated with TGF-β. (**A**) Western blot results demonstrating the increase of PDGFR-α, collagen I, and α-SMA synthesis in the PHF14-knockdown group. Anti-GAPDH was used to verify equivalent loading. (**B**–**D**) Semiquantitative analysis of PDGFR-α, collagen I, and α-SMA protein abundance in the TGF-β-treated cells. (**E**) The schematic diagram of the PDGFR-α promoter region and the primer design. (**F**) Chromatin immunoprecipitation of the PDGFR-α promoter region with anti-FLAG antibody. *P < 0.05, compared with scrambled controls (n = 3). GAPDH, glyceraldehyde 3-phosphate dehydrogenase; PDGFR-α, platelet-derived growth factor receptor-α; NC, negative control; PC, positive control; P1–P4, primers 1 to 4; TSS, transcription start site.

Previous research has identified PHF14 as a negative regulator of mesenchymal cell proliferation via suppression of PDGFR-α transcription, and the PHF14-containing complex binding site has been found to bind to the promoter of PDGFR-α in mouse embryonic fibroblasts through ChIP assays^14^. We briefly validated these findings in the NRK-49F cell line. Knockdown of PHF14 in NRK-49F cells enhanced not only collagen I and α-SMA synthesis but also PDGFR-α expression (Fig. 5A–D) under conditions of TGF-β stimulation. Since the PHF14/PDGFR-α promoter potential binding regions in mouse mesenchymal cells have been revealed by ChIP analysis^14^, we performed sequence alignment and designed four pairs of primers accordingly (Fig. 5E) to validate the binding activity in NRK-49F cells. By phf14-3-FLAG adenovirus infection, exogenous PHF14 was introduced into NRK-49F cells. ChIP analysis using anti-FLAG revealed the binding site of PHF14 to be in the upstream region of the PDGFR-α transcription start site (Fig. 5F), which suggested that PHF14 might function in a similar way in renal fibroblasts as in mouse embryonic fibroblasts. PHF14 directly repressed the transcription of PDGFR-α in renal fibroblasts, thus suppressing the PDGF pathway signaling overactivation.

### High recombination efficiency of Cre-ER^TM^; phf14 flox/flox adult mice

**Figure 6:**
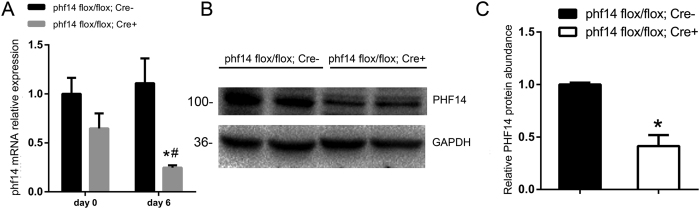
Tamoxifen-induced PHF14 knockout in adult mouse kidneys. (**A**) Quantitative PCR of phf14 in the control group (phf14 flox/flox Cre−) and the phf14-knockout group (phf14 flox/flox Cre+) before (day 0) and after five consecutive days (day 6) of an intraperitoneal tamoxifen injection. *P < 0.05, compared with controls at day 6; ^#^*P* < 0.05, compared with mice in the knockout group at day 0. (**B**) Western blot results showing the decrease of PHF14 protein expression in phf14 flox/flox Cre+ mice after tamoxifen administration, compared with control mice. (**C**) Semiquantitative analysis of PHF14 protein abundance in kidneys of phf14 flox/flox Cre+ mice and phf14 flox/flox Cre− mice (n = 5).

The recombination efficiency of the phf14 locus in the kidney of Cre-ER^TM^, phf14 flox/flox mice was verified by quantitative PCR and western blot experiments. After tamoxifen administration for 5 days, PHF14 expression was effectively suppressed in the kidney (Fig. 6A–C) and other major organs like the liver and lung (data not shown).

### phf14 deletion in adult mice exacerbates renal fibrosis following folic acid-induced renal injury

**Figure 7:**
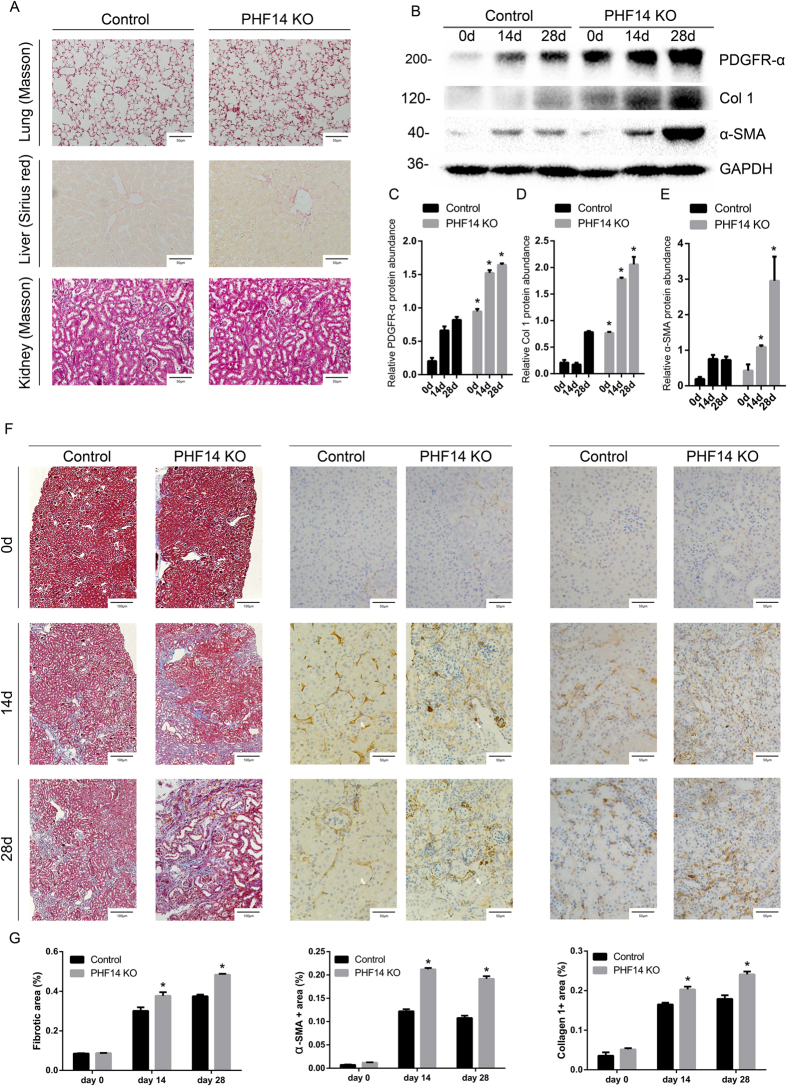
phf14 deletion in adult mice exacerbated renal fibrosis following folic acid-induced renal injury. (**A**) Morphological analyses of lung, liver, and kidney tissues from PHF14-knockout mice compared with those from phf14 flox/flox Cre− mice on the 28th day after tamoxifen administration for 5 days. (**B**) Western blot results showing increased collagen I and α-SMA expression levels in the PHF14-knockout mice at the indicated time points (day 14 and day 28) after folic acid injection, compared with phf14 flox/flox Cre− mice. Anti-GAPDH was used to verify equivalent loading. (**C**–**E**) Semiquantitative analysis of collagen I, α-SMA, and PDGFR-α protein abundance in the kidneys. *P < 0.05, compared with sham controls (n = 5). (**F**) Representative Masson and immunohistochemical staining assay images demonstrating the severity of renal fibrosis in the PHF14-knockout mice on day 14 and day 28 after folic acid administration, compared with the sham control. (**G**) Quantitative analysis of fibrotic area/α-SMA-positive area/collagen I-positive area based on Masson staining and immunohistochemical staining. *P < 0.05, compared with the corresponding time point in the controls (n = 5). KO, knockout; Col 1, collagen I; PDGFR-α, platelet-derived growth factor receptor-α; α-SMA, α-smooth muscle actin; GAPDH, glyceraldehyde 3-phosphate dehydrogenase.

In previous studies, unconditional knockout of PHF14 was neonatally lethal due to the role of PHF14 in organogenesis^14^,^17^; while in conditional knockout mouse models, all the PHF14 flox, Cre+ mice were born normal. Over the 28-day observation period after tamoxifen-induced recombination, adult mice with PHF14 knockout did not show obvious changes in their behavior, feeding, or appearance. Moreover, morphological analyses of the livers (Sirius red staining), lungs, and kidneys (Masson staining) did not demonstrate obvious interstitial extracellular matrix (ECM) accumulation or fibrosis in PHF14-knockout adult mice (Fig. 7A).

After a subsequent pro-fibrotic insult (folic acid administration), the kidneys showed more severe fibrotic pathological changes by Masson staining in conditional PHF14-knockout mice (Fig. 7F). Expression of PDGFR-α, collagen I, and α-SMA was increased in the kidneys of PHF14-knockout mice, compared with control littermates (Fig. 7B–E). In addition, the section areas with positive staining for collagen I and α-SMA, which was mostly localized in the renal interstitium in both groups, were larger in PHF14-knockout mice than in control mice (Fig. 7F–G). Taking the evidence together, PHF14 might not perform its regulatory function under physiological conditions at the adult stage, and PHF14 knockout in adult mice did not result in pathological and physiopathological events over a relatively long period. While during the progression of fibrosis after the pro-fibrotic insult, PHF14 played an important role in the regulation of renal tissue repair and regeneration; and these processes could not function properly when PHF14 expression was lacking. As shown by the studies, failed wound healing was the major event underlying progressive TIF.

## Discussion

The formation of renal fibrotic lesions after various insults is modulated by multiple mediators. Among the complex mediator network, the central role of TGF-β has been widely recognized^22^,^23^,^24^,^25^. Emerging studies have revealed the underlying TGF-β downstream pathways and negative feedback regulatory routes during the progression of fibrosis^24^,^26^,^27^,^28^. As a potent factor that negatively regulates the pro-fibrotic effect of TGF-β, PHF14 expression is activated in fibrotic kidneys, which is mediated by TGF-β/smad3 signaling itself. The elevated PHF14 expression may hinder the process of renal fibrogenesis by suppressing PDGFR-α gene transcription. PHF14 deletion in mice promotes ECM production and renal fibrosis after folic acid administration. This research provides novel mechanistic insight into the self-limitation of TGF-β-dominated renal pro-fibrotic signaling. To the best of our knowledge, this is the first study to introduce PHF14 as the mediator for re-balancing the biological route for pro-/anti-fibrotic regulators. The results are helpful for us to understand the interactions of the factors in the fibrogenesis signaling network.

It is well documented that TGF-β can mediate renal fibrosis in several ways^29^. This cytokine directly leads to ECM accumulation^30^ and stimulates the transdifferentiation of several types of cells toward myofibroblasts^31^,^32^. In addition, TGF-β can also induce the expression of other cooperative profibrotic cytokines such as PDGF and epidermal growth factor^33^. TGF-β exerts its biological function mainly through smad signaling, and members of the smad family play distinct roles in renal fibrosis. Briefly, smad3 has been proven to play a pivotal pathogenic role; while smad7, as an inhibitory member of the smad superfamily, antagonizes the action of smad3^34^,^35^. Numerous known factors related to tissue repair and tissue homeostasis are regulated in a TGF-β/smad-dependent manner^36^,^37^. Based on the abovementioned knowledge, therapeutic approaches that involve blocking the pathogenic action of TGF-β on multiple levels have been developed to control the progression of renal fibrosis. Preclinical research on some of these promising interventions has been undertaken^38^,^39^,^40^; however, few studies have progressed to clinical trials. Unacceptable adverse effects induced by complete TGF-β inhibition were the major concerns^41^,^42^. In this study, we revealed that PHF14, which is upregulated in a smad3-dependent manner, is a mediator for re-balancing the biological route for pro-/anti-fibrotic regulators. Theoretically, therapeutic approaches targeting PHF14-related pathways, including upstream or downstream mediators, may avoid the adverse effects caused by systemic TGF-β inhibition but keep the renal protective effect. Our findings may provide the rationale for developing potential novel therapies for kidney fibrosis. After pro-fibrotic insults, upregulated TGF-β signaling activates the PDGF/PDGFR pathway, and the latter will aggravate renal fibrosis^43^. Solid evidence indicates that PHF14 negatively regulates mesenchyme growth by acting as a transcription factor to suppress PDGFR-α expression^14^,^17^. We briefly validated the previous findings in a kidney fibrotic model.

Renal tubular epithelial cells have been suggested to play an active role in kidney fibrosis via multiple biological processes including epithelial–mesenchymal transition^44^. Whether there exist other downstream factors of PHF14 to regulate mesenchyme growth and whether renal tubular epithelial cells participate in the regulation of fibrosis via PHF14 await further studies.

In previous studies on PHF14, it was found that PHF14 is required to regulate mesenchyme growth in the process of embryonic organ formation. Mice with unconditional knockout of PHF14 die just after birth due to respiratory failure, and uncontrolled mesenchyme growth in major organs (including the lungs, liver, and kidneys) has been revealed by pathological analysis^14^,^17^. To investigate the biological function of PHF14 in adult animals, tamoxifen-inducible PHF14-knockout mice (CAGGCre-ER^TM^+) were used to delete phf14 at 8 weeks after birth. According to our results, over the 28-day observational period, a lack of PHF14 expression in adult mice did not result in pathological or functional changes in major organs, suggesting that PHF14 did not perform a regulatory function under physiological conditions in adult mice. Besides, the intact function of major organs after PHF14 knockout avoided the interference from abnormal systematic conditions in subsequent experiments.

In summary, PHF14 expression was upregulated persistently during the progression of renal fibrosis after acute kidney injury, and the upregulation was induced by TGF-β/smad3 signaling. The elevated PHF14 levels hindered the process of renal fibrogenesis by supressing PDGFR-α expression. Therefore, the TGF-β/smad3/PHF14 pathway can be regarded as a self-limiting mechanism of TGF-β-dominated renal pro-fibrotic signaling and provides re-balancing of the biological route for pro-/anti-fibrotic regulators.

## Methods

### Cell culture and treatment

Rat kidney interstitial fibroblasts (NRK-49F cells) were obtained from the American Type Culture Collection (Manassas, VA). NRK-49F cells were cultured in Dulbecco’s modified Eagle medium–Ham’s medium (Gibco/Life Technologies, Grand Island, NY) supplemented with 10% fetal bovine serum (Gibco/Life Technologies).

### siRNA inhibition of PHF14 and exogenous expression of PHF14

NRK-49F cells were transfected with 50 nM phf14 siRNA or scrambled negative control siRNA (Biomics Biotechnologies Co., Ltd., Shanghai, China) for 36 h using Lipofectamine 2000 reagent (Invitrogen, Grand Island, NY), according to the manufacturer’s instructions. The siRNA sequences used were as follows: sense, 5′-GAU GGA ACC AAA CGA UCA A-3′ and anti-sense, 5′-UUG AUC GUU UGG UUC CAU C-3′. The expression of phf14 was determined by quantitative PCR. Stable cell lines were treated with TGF-β1 to induce fibrotic responses. At least three independent experiments were performed. For viral infection experiments, 50 pfu/cell of adenovirus encoding Phf14-3-FLAG or green fluorescent protein control were used.

### ChIP analysis

ChIP analysis was performed by an Enzymatic Chromatin Immunoprecipitation kit (Cell Signaling Technology, Beverly, MA), according to the manufacturer’s instructions. In brief, NRK-49F cells were first cross-linked with 1% formaldehyde for 10 min at room temperature and then quenched with glycine. Chromatin digestion was conducted to generate 150- to 900-bp DNA fragments using Micrococcal Nuclease. Immunoprecipitation was performed with p-smad3 antibody (Cell Signaling Technology) or FLAG-antibody (Sigma-Aldrich, St. Louis, MO); normal isotype IgG was used as a negative control, and anti-H3 antibody was used as a positive control. Precipitated DNA was detected by PCR using predesigned specific primers (Table 1).

### Animals

From the Jackson Laboratory, wild-type C57BL/6 J mice (stock no.: 000664) and tamoxifen-inducible Cre− expressing CAGGCre-ER^TM^ mice (stock no.: 004682) were obtained. The generation of phf14 flox/flox mice has been reported previously by us^17^. All animal protocols were conducted in accordance with guidelines for the Care and Use of Laboratory Animals by the National Research Council and were approved by the Animal Care and Use Committee at the Second Military Medical University. All experiments were performed in accordance with the approved relevant guidelines and regulations. Adult mice (8–12 weeks old) weighing 25–35 g were used in this study.

### Genotyping and PHF14 deletion

Genotyping was performed by tail DNA PCR analysis. Tail DNA was isolated using a Mouse Direct PCR Kit (Biomake, Houston, TX, USA). The primer sequences used for genotyping were as follows: floxed phf14 allele forward, 5′-CTA TTT TCT TGA TTA TAG ATG CAG-3′ and floxed phf14 allele reverse, 5′-GCC TTC TAA GTT CCA GCT ACT AG-3′ CAGGCre-ER^TM^ forward, 5′-ATT GCT GTC ACT TGG TCG TGG C-3′ and CAGGCre-ER^TM^ reverse, 5′-GGA AAA TGC TTC TGT CCG TTT GC-3′. The PCR products were analyzed by agarose gel electrophoresis. Mice with genotype phf14 flox/-, CAGGCre-ER^TM^+ were created by crossbreeding phf14 flox/flox with CAGGCre-ER^TM^+. The resulting phf14 flox/-, CAGGCre-ER^TM^+ mice were backcrossed to phf14 flox/flox to obtain phf14 flox/flox, CAGGCre-ER^TM^+ mice. In addition, their littermates with genotype phf14 flox/flox, CAGGCre-ER^TM^- were used as controls. Adult phf14 flox/flox, CAGGCre-ER^TM^+ mice and controls of the same age were intraperitoneally injected with tamoxifen (Sigma-Aldrich) (3 mg/40 g body weight) in corn oil (Sigma-Aldrich) vehicle for five consecutive days as suggested elsewhere to induce the expression of Cre^45^.

### Folate model of acute renal injury and chronic nephropathy

A single intraperitoneal injection of folic acid (250 mg/kg body weight) in 0.3 M NaHCO_3_ vehicle or vehicle alone was administered to adult mice as reported elsewhere^46^. For construction of the mouse model of renal fibrosis, folic acid or vehicle was injected into mice at 10 weeks after birth; for kidney insults to PHF14-conditional knockout mice, folic acid or vehicle was injected on the day following tamoxifen administration for 5 days. Mice were euthanized at 2 days (acute kidney injury phase), 7 days, 14 days, or 28 days after folic acid injection, and the kidneys were collected from folic acid-treated or vehicle-treated animals.

### Morphological analyses

Kidneys were fixed in 4% paraformaldehyde and embedded in paraffin. Paraffin sections (4-μm thick) were stained with Masson’s trichrome. Percentages of fibrotic areas were quantified using the National Institutes of Health (NIH) ImageJ program.

### Immunohistochemical and immunofluorescence staining

Kidneys were fixed in 4% paraformaldehyde and embedded in paraffin. Four-μm-thick sections of paraffin-embedded kidney tissue were subjected to immunohistochemical staining with anti-PHF14 (Abcam, Cambridge, MA), anti-α-SMA (Abcam), or anti-collagen I (Santa Cruz, Dallas, TX) antibodies. For cultured cells, after fixing and rinsing, specimens were blocked with blocking buffer (5% normal serum/phosphate-buffered saline/0.3% Triton^TM^ X-100) and incubated with the primary antibodies overnight at 4 °C, followed by incubation with fluorochrome-conjugated secondary antibodies. Cells were counterstained with 4′,6-diamidino-2-phenylindole (DAPI) to visualize the nuclei. Images were obtained with the use of a microscope (Olympus BX51, Tokyo, Japan). The percentages of positively stained areas by immunohistochemistry were quantified using the NIH ImageJ program.

### RNA extraction and real-time PCR examination

Total RNA was isolated from the cultured cells and kidney tissues using Trizol reagent (Takara, Shiga, Japan), according to the manufacturer’s instructions. cDNA was synthesized with 1 μg of total RNA, cDNA synthesis mix (Biomake), and oligo (dT) primers. The primers used in this study are listed in Table 1. Gene expression was measured by a real-time PCR assay (Biomake) and a 7900HT real-time PCR system (Applied Biosystems, Foster City, CA). The relative amount of mRNA to the internal control was calculated as 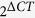, in which 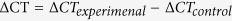.

### Western blot analyses

Cell or tissue lysates were prepared in RIPA buffer (50 mM Tris HCl, 150 mM NaCl, 1 mM EDTA, 2% sodium dodecyl sulfate (SDS), 1% Triton, and phosphatase and protease inhibitors) and clarified by centrifugation. Equal amounts of protein were run on SDS-polyacrylamide gels under reducing conditions, transferred onto polyvinylidene difluoride membranes, blocked in 3% bovine serum albumin, and incubated with various primary antibodies. Appropriate secondary antibodies were used before development with enhanced chemiluminescence reagent. The primary antibodies used were as follows: phospho-Smad2/3, total Smad2/3, and PHF14 (Abcam); α-SMA, collagen I, and PDGFR-α (Cell Signaling Technology). Anti-GAPDH (Sigma-Aldrich) served as a loading control.

### Statistical analyses

All data examined were presented as the mean ± standard error. Comparisons between two groups were conducted using the two-tailed *t* test when the data were normally distributed and Wilcoxon tests for skewed continuous data. *P* < 0.05 was considered statistically significant. All analyses were carried out using SPSS 12.0 (SPSS Inc., Chicago, IL), and all graphs were generated using GraphPad prism software (GraphPad Software, Inc.).

## Additional Information

**How to cite this article**: Yang, B. *et al*. PHF14: an innate inhibitor against the progression of renal fibrosis following folic acid-induced kidney injury. *Sci. Rep.* **7**, 39888; doi: 10.1038/srep39888 (2017).

**Publisher's note:** Springer Nature remains neutral with regard to jurisdictional claims in published maps and institutional affiliations.

## Figures and Tables

**Table 1:** Primers used for PCR.

Gene name	Forward	Reverse
Primers used for quantitative PCR		
gapdh (mouse)	5′-GTCTTCACCACCATGGAGAAGG-3′	5′-CTAAGCAGTTGGTGGTGCAGGA-3′
gapdh (rat)	5′-AAGGTGGTGAAGCAGGCGGC-3′	5′-GAGCAATGCCAGCCCCAGCA-3′
phf14 (mouse)	5′-GCTTCTCTCCTGGAAGCTCTTGATT-3′	5′-ATCACTACAGGAACCTTCTCCACTG-3′
phf14 (rat)	5′-CACAGATACAAATGGCTAAA-3′	5′-AGAACCAGAGGACGAAAG-3′
α-SMA (mouse)	5′-GTGACATCGACATCAGGAAAGA-3′	5′-GATCCACATCTGCTGGAAGG-3′
α-SMA (rat)	5′-CCACTGCTGCTTCCTCTTCTTC-3′	5′-GCCCGCCGACTCCATTCC-3′
collagen Ia (mouse)	5′-TGACTGGAAGAGCGGAGAGTA-3′	5′-GACGGCTGAGTAGGGAACAC-3′
collagen Ia (rat)	5′-CGAGTATGGAAGCGAAGG-3′	5′-AGTGATAGGTGATGTTCTGG-3′
pdgfr-α (mouse)	5′-CTTCGGAAGAGAGTGCCATC-3′	5′-CACCAGGTCCGAGGAATCTA-3′
pdgfr-α (rat)	5′-AATGAAGGTGGCTGTGAAGATGC-3′	5′-AGATGCGGTCCCAAGTGAGTC-3′
Primers for the upstream promoter region of phf14 used for chromatin immunoprecipitation		
1	5′-GCTCACAACTGTCTGTAACTCC-3′	5′-GGTTAGGGTGTTTGATGTGCTTG-3′
2	5′-CACAGACGTACAAGCACATCAA-3′	5′-TCTATACCCTCCCACCACCT-3′
3	5′-CACTGAAGCAGTAGGTGGTG-3′	5′-AGACAATAGGGAGAAGAACG-3′
4	5′-GGGTCGTTCTTCTCCCTATTGT-3′	5′-CACTTCCCCTGCGCTCCCTGGC-3′
Primers for the upstream promoter region of pdgfr-α used for chromatin immunoprecipitation		
1	5′-GGTGGAGCCAATACTCAATG-3′	5′-TCCCAGTTTCAGACTCAGAG-3′
2	5′-CTGGGAGTTATTCTGAAATGG-3′	5′-CTTGAGCATCTTCTGTTCTG-3′
3	5′-GGGGTTGAATGGGATTCTGAC-3′	5′-TCCATAGAGAGGGTCTTCCAATC-3′
4	5′-TGGAGTTGCTCTCACAC-3′	5′-TCACTGCCTTTGTTTATATT-3′

PCR, Polymerase chain reaction.
