# Author Correction: PHF14: an innate inhibitor against the progression of renal fibrosis following folic acid-induced kidney injury

**DOI:** 10.1038/s41598-020-62765-7

**Published:** 2020-04-02

**Authors:** Bo Yang, Sixiu Chen, Ming Wu, Lin Zhang, Mengna Ruan, Xujiao Chen, Zhengjun Chen, Changlin Mei, Zhiguo Mao

**Affiliations:** 1 Division of Nephrology, Kidney Institute of CPLA, Changzheng Hospital, Second Military Medical University, Shanghai, 200003 People’s Republic of China; 2 School of Life Science and Technology, Shanghai Tech University, Shanghai, 200031 People’s Republic of China; 3 State Key Laboratory of Molecular Biology, Institute of Biochemistry and Cell Biology, Shanghai Institutes for Biological Sciences, Chinese Academy of Sciences, Shanghai, 200031 People’s Republic of China

Correction to: *Scientific Reports* 10.1038/srep39888, published online 03 January 2017

This Article contains an error in the Acknowledgements section.

“This work was supported by the National Nature Science Fund of China (No. 81000281, 31430055, 31190062, and 31170723), Chinese Society of Nephrology (No. 13030340419), Major Fundamental Research Program of Shanghai Committee of Science and Technology (No. 12DJ1400300), and Key Projects in the National Science & Technology Pillar Program in the Twelfth Five-year Plan Period (No. 2011BAI10B00).”

should read:

“This work was supported by the National Natural Science Foundation of China (No. 81570621, 81000281, 31430055, 31190062, and 31170723), Chinese Society of Nephrology (No. 13030340419), Major Fundamental Research Program of Shanghai Committee of Science and Technology (No. 12DJ1400300), and Key Projects in the National Science & Technology Pillar Program in the Twelfth Five-year Plan Period (No. 2011BAI10B00).”

